# ETHNIC AND NEIGHBORHOOD DIFFERENCES IN POVERTY AND DISABILITY AMONG OLDER ASIAN AMERICANS IN NEW YORK CITY

**DOI:** 10.1093/geroni/igac059.1092

**Published:** 2022-12-20

**Authors:** Keith Chan, Christina Marsack-Topolewski

**Affiliations:** Hunter College, City University of New York, New York, New York, United States; Eastern Michigan University, Ypsilanti, Michigan, United States

## Abstract

Asian Americans are the fastest growing and aging U.S. population, and occupy both extremes of socioeconomic and health indices. Using the 2016 NYC.gov dataset, multilevel logistic regression analyses were conducted to examine the relationship of poverty, acculturation and neighborhood-level variables with disability for different ethnic groups of Asian older adults (Chinese, South Asian, Filipino, Japanese, Korean and Vietnamese) in New York City. Findings indicated that South Asian older adults had higher odds for disability compared to other ethnic groups. Living in a neighborhood with higher percentages of persons of the same ethnicity was protective for Chinese older adults only. There is an important opportunity for interprofessional collaborations through education, awareness, screening and intervening to enhance systems of care for Asian older adults. Social workers can play a pivotal role in providing key linkages to form interprofessional solutions and shared efforts to address the needs of this understudied and under-resourced population.

